# The impact of self-regulated learning strategies on academic performance for online learning during COVID-19

**DOI:** 10.3389/fpsyg.2022.1047680

**Published:** 2022-11-29

**Authors:** Ling Xu, Peng Duan, Shirley A. Padua, Chengyou Li

**Affiliations:** ^1^School of Business, Liaocheng University, Liaocheng, China; ^2^School of Education, The Philippine Women's University, Manila, Philippines; ^3^School of Computer Science, Liaocheng University, Liaocheng, China

**Keywords:** self-regulated learning strategies, online learning, metacognition, academic performance, college students

## Abstract

The COVID-19 pandemic led higher education institutions to transition to online learning. The present study was designed to investigate students' self-regulated learning strategies on academic performance in online learning. We analyzed the differences in college students' self-regulated learning (SRL) strategies according to their grade point average (GPA). The study included 1,163 students at a distance education university in China. Two online questionnaires were used to determine online SRL strategies. GPA scores were obtained from the university exam database to determine academic performance. The analysis showed that there are great differences between different self-regulated strategies and between different students when accepting the online learning. The analysis also showed that self-evaluation, metacognitive self-regulation, and effort regulation were positive predictors of academic progress, besides, self-evaluation and effort regulation had mutual influence effect on the improvement of GPA in online learning. These data will help teachers, education policymakers, and education administrators adopt and implement online learning services to improve students' academic performance.

## 1. Introduction

In January 2020, when the COVID-19 pandemic broke out, many changes were made in various sectors of life, including education. In response to the impact of the COVID-19 pandemic on the field of education, many universities transitioned to remote learning where classes were held online and the teaching mode changed to online teaching, which has completely changed the learning mode of college students (Crawford et al., 2020). In the online learning mode, students and teachers lack face-to-face interaction and communication as they did in a classroom, and it is difficult to perceive the micro-changes of each other's expressions and actions in the process of communication. Online learning relies on the use of asynchronistic and synchronistic interaction within a virtual environment (Serdyukov, 2020). Further, students who study online have more opportunities to learn information and access learning resources flexibly and autonomously instead of studying completely according to the teacher's guidance (Waha and Davis, 2014). In the learning process, teachers are no longer the leaders and regulators of learning, but they truly become participants and instructors of learning. All in all, this is a new learning mode characterized by flexible teaching and active learning (Nikolaki et al., 2017; Nerantzi, 2020). Many studies have shown that academic success in an online learning environment requires high level of self-regulated learning (SRL) skills and great ability to control the learning process (Broadbent and Poon, 2015; Jia, 2021). Thus, self-regulation becomes a crucial factor for successful online learning.

A variety of definitions have been used to describe SRL. Zimmerman posited that SRL is “the degree to which students are meta-cognitively, motivationally, and behaviorally active participants in their own learning process” (Zimmerman, 1990) which has been the most widely accepted definition. SRL is generally described as a cyclical process, often triggered by the formulation of goals and the subsequent employment of strategies, followed by engagement in reflection and the formulation of new learning goals (Zimmerman, 2002). In addition, students' SRL ability is dynamically developed, and the learning environment can influence the way students learn (Chen and Bonner, 2020). In other words, the same learning strategy usually works differently in different environments (Broadbent and Poon, 2015). Examining SRL strategies in the online learning environment is important given that this environment has been noted as requiring individuals to be more autonomous in their learning.

The rapid development of online learning during the pandemic led to the accumulation of online learning data that provide a basis for quantitative research on online learning. However, our understanding of teaching and learning in this new environment is relatively lacking. Most studies about online learning focus on the weak online teaching facilities, the adverse impact of home environment, the adjustment of students' thinking styles, the degree of cohesion of curriculum design, and the sense of online learning efficacy (Carter et al., 2020; Pokhrel and Chhetri, 2021; Ulfatun et al., 2021). Sintema et al. found students' levels of academic performance are likely to decline due to reduced time spent engaging with learners and a lack of counseling with teachers when they experience online learning (Sintema, 2020). However, SRL ability is to accurately analyze and choose appropriate methods and strategies to adapt to the new environment and achieve academic success in the face of such environmental changes (Araka et al., 2020). Most researchers believe that self-regulation is a context-specific process (von Suchodoletz et al., 2015). Effective implementation of learner support requires an understanding of which SRL strategies are most effective in online learning environment. Therefore, it is very important to investigate the academic performance and analyze the SRL capabilities and strategies under a passive online learning mode during the pandemic, which can provide a useful reference and insight for future teaching and learning changes.

Thus, this study aims to analyze the differences and associations in SRL strategies based on their academic performance, to understand how students could best apply SRL strategies to achieve academic progress within the online environment.

### 1.1. Academic performance in online learning environments

Academic performance can be generally defined as achieving a particular result in an exam or subject and is ordinarily expressed in terms of a numerical grade or grade point average (GPA) (Richardson et al., 2012). The higher the GPA, the better the students' academic performance. In the most study of predicting academic performance for online learning, the theory of SRL has been used as theoretical grounding (You, 2016). The prediction model based on SRL theory proposed that students who manage their time appropriately, persevere in understanding the learning material were more likely to achieve higher grades in the online learning (Broadbent and Poon, 2015). For example, Kizilcec et al. found that task management strategies, such as reserving time in the week for studying, starting and finishing a chapter on the same day, working with others on the course, having clear objectives and planning around those goals, and applying what one has learned were helpful for online learning (Kizilcec et al., 2017). Muilenburg et al. also found statistically significant relationship between age, gender, or ethnicity (Muilenburg and Berge, 2005). At the same time, some studies have found an increase in students' academic performance in emergency remote teaching (Iglesias-Pradas et al., 2021). Although some measures show a positive impact on achieving academic success, there is a gap about how COVID-19 (stay-at-home) measures and online learning affects students' academic performance.

A large number of researchers have shown that a positive association exists between SRL strategies and college students' academic outcomes (Nota et al., 2004; Kramarski and Gutman, 2006; Beishuizen and Steffens, 2011; Richardson et al., 2012). The application of SRL strategies typically predicts high academic achievement (Broadbent and Poon, 2015). Students' SRL in an online learning environment specifically manifests their ability to develop their own learning goals, formulate the entire learning plan in advance, effectively use learning content and materials, be effective in the learning process, and regulate and conduct a self-reflective assessment of learning results (Artino, 2007). The unique feature of online learning is that it gives students control over when, what, and how to study to a great extent (Dumford and Miller, 2018). As the online learning environment is characterized by autonomy, self-regulation becomes a critical factor for success in online learning. Researchers have suggested that SRL is of greater importance in online learning environments due to its autonomous nature (Wong et al., 2019).

### 1.2. SRL strategies and academic performance

A widely accepted classification of SRL strategies was first described by Pintrich as follows: rehearsal, elaboration, organization, critical thinking, metacognitive self-regulation, time and study environment, effort regulation, peer learning, and help-seeking (Pintrich et al., 1991). In the student's learning process, these strategies interact with one another in each cycle, changing the student's SRL skills and strategies (Bandura and Cervone, 1986). As SRL strategies that students perform, these SRL strategies were a function of an individual's desire to achieve in their learning and necessary for the success of learning in the online environment. From the perspective of social cognition, the development of SRL skills and strategies is affected by the interaction of personal, behavioral, and environmental factors, in the form of reciprocal causality (Zimmerman and Bandura, 1994; Zakiah and Fajriadi, 2020). Thus, certain strategies may be more effective in certain environments than others (Ashley and Tuten, 2015).

The role of learning strategies in gaining academic success has been widely investigated for campus-based college students. In 2012, Richardson, Abraham, and Bond conducted a retrospective study and meta-analysis of the relationship between college students' learning strategies and GPA. The results showed that effort regulation was the most important learning strategy positively correlated with academic outcomes, followed by time and learning environment management and metacognitive self-regulation, whereas rehearsal, elaboration, and organization had the least empirical support (Richardson et al., 2012; Broadbent and Poon, 2015). The development of SRL is an active process. With the changes and development of students' learning environment, SRL strategies will inevitably change as well. Research has found that the most powerful SRL strategies are metacognition, time management, and effort adjustment (Richardson et al., 2012). Broadbent and Poon found that four learning strategies including metacognition, time management, effort regulation, and critical thinking were significantly associated with online learner's grades (Broadbent and Poon, 2015). To sum up, the existing literature showed that in an online learning environment, SRL strategies were related to students' academic performance. However, due to the different characteristics of each learning strategies and the different challenges faced by students, the effect of the SRL strategies involved in predicting academic performance may be different in different environments. Thus, the exploration of the predictors of online learning success, the effect of different strategies on academic performance and the effectiveness of different SRL strategies under the online learning model during the pandemic is becoming increasingly important.

## 2. Methods

### 2.1. Study design

This study was based on longitudinal GPA data collected from 1,163 college students in China. The Online Self-regulated Learning Questionnaire (OSLQ) and Motivated Strategies for Learning Questionnaire (MSLQ-B) were used to include SRL strategies. Before investigating the questionnaires, G-power was used to budget the sample size. The effect size *t* = 0.8, α = 0.001, and the power *P* = 0.95 were selected. The calculated sample size and the total sample size were 73 and 146, respectively. The number of valid questionnaires were 388, which can reach the priori sample size. The questionnaire items were written in English and translated into Chinese. To ensure cross-linguistic equivalence, the results were independently translated back to English by two English professors.

### 2.2. Participants

All participants were from a public university located in Shandong Province, China. From March to July 2020, due to the impact of COVID-19, universities generally adopted an online teaching model. Undergraduates receiving online lectures are our research objects. We recruited 1,163 undergraduates enrolled in online learning during the second term of the 2019–2020 school year. By comparing their GPA obtained in three semesters, we selected a total of 410 students who had significantly increased and decreased GPA in the online learning mode. We posted an online questionnaire, and 406 students volunteered to be part of the study and completed the online survey (the efficiency recall rate was 99.02%). In order to ensure the quality of the questionnaire, we gathered students to fill in the questionnaire in the classroom. Before answering the questionnaire, we carefully explained to the students the purpose of the questionnaire, the notes for filling in and the expectation of getting real feedback from everyone. After receiving the questionnaire, we calculated the average response time and set the upper and lower limits of time according to the mean ± 2 standard deviation. We finally got 388 high-quality questionnaires and about 4.4% of the extreme duration samples were screened out. Among the 388 students, 54.12% (*n* = 210) were female, and 45.88% (*n* = 178) were male. As for their age, 100% (*n* = 388) of the students were aged 18–22, and their average age was 19.74 years old (*SD* = 0.53).

### 2.3. Data collection

The students' GPA scores were obtained from the scoring system. The research conducted online questionnaires to investigate the SRL strategies of students with fluctuating GPA. The students completed the questionnaires to measure their SRL ability and strategies. All students were briefed about the purpose of this study before answering the questionnaire. In addition, we obtained the approval of the ethics committee of Liaocheng University. The participants filled out the surveys voluntarily with no negative consequences for not filling them. The questionnaire items were designed in English and translated into Chinese.

### 2.4. Online SRL

SRL is a process that changes as the learning environment changes. With the change and development of students' learning environment, the measurement method of SRL is bound to change. Therefore, the OSLQ developed by Barnard was used in this study to measure students' SRL level in online learning (Barnard et al., 2009). The OSLQ measures the skills that involve the various processes in the regulation of cognitive and metacognitive aspects, motivation, and behavior. The OSLQ is a means of assessing individuals' SRL in online learning situations by asking about their cognitive and metacognitive strategies for learning (Amir and Kamal, 2011). This scale has 24 items measuring six dimensions: environment structuring (four items), goal setting (five items), time management (three items), help-seeking (four items), task strategies (four items), and self-evaluation (four items). The responses are rated on a 5-point Likert scale ranging from 1 (none at all) to 5 (a great deal). Barnard et al. demonstrated that the OSLQ had good reliability and validity in both online and hybrid environments in two separate studies (Barnard et al., 2009). We employed confirmatory factor analysis (CFA) to test all variables' construct validity and used the standards for good fit to determine whether the variable had good structural validity (Hu and Bentler, 1998): *X*^2^/*df* < 5, root mean square error of approximation (RMSEA) <0.1, and comparative fit index (CFI) > 0.90. The online SRL indicators of CFA were *X*^2^/*df* = 3.27, RMSEA = 0.08, and CFI = 0.93.

### 2.5. SRL strategies

To assess students' SRL strategies, we administered the MSLQ-B, which is a self-report survey. Originally, the MSLQ included 81 items separated into two categories: motivation and learning strategies. Because we were only concerned with the learning strategies, a learning strategy questionnaire was used in this study to determine students' SRL strategies. The learning strategies include 31 items regarding students' use of different cognitive and metacognitive strategies and 19 items concerning students' management of different resources (Pintrich et al., 1991). The subdimensions of learning strategies were rehearsal (four items), elaboration (six items), organization (four items), critical thinking (five items), metacognitive self-regulation (twelve items), time and study environment (eight items), effort regulation (four items), peer learning (three items), and help-seeking (four items). The MSLQ-B makes use of a 5-point Likert scale ranging from 1 (strongly disagree) to 5 (strongly agree). CFA results showed that the SRL strategies fit indices were *X*^2^/*df* = 4.54, RMSEA = 0.09, and CFI = 0.93.

### 2.6. Analysis procedures

The current study explored the students' SRL strategies based on their GPA in online learning mode. The GPA was obtained in score system with the consent of the manager. Descriptive analysis and questionnaire reliability analysis including all demographic variables and the subscale scores of the OSLQ and MSLQ-B were completed in SPSS 22.0. The subscale scores from the OSLQ and MSLQ-B for the GPA-up and GPA-down students were analyzed by SPSS 22.0 (independent-sample *t*-test). Multivariate analysis of variance and influence effect were conducted in SPSS 22.0. *p* < 0.05 was considered a significant difference. All graphics were drawn using GraphPad Prism software. The data used to support the findings of this study are available from the corresponding author upon request.

### 2.7. Ethics statement

We obtained the approval from the ethics committee of Liaocheng University. The participants completed the surveys voluntarily with no negative consequences for not completing them. All participants gave written informed consent following the Declaration of Helsinki.

## 3. Results

### 3.1. Descriptive analysis of the OSLQ and MSLQ-B

To explore the SRL situation of students with different academic performance in the online learning mode, we screened out 410 students with | GPA changes | > 1.0 in online learning mode. We posted OSLQ and MSLQ-B online questionnaire, and finally got 388 high-quality questionnaires. Among the 388 students with the highest GPA changes, 202 GPA-down students and 186 GPA-up students were identified. At the same time, we conducted questionnaires on the status of online SRL strategies of these 388 students. The reliability of the OSLQ and MSLQ-B was calculated using Cronbach's α coefficient. Cronbach's α for the measure of OSLQ and MSLQ-B was 0.96 and 0.97, respectively, which demonstrated that the internal consistency of the scale was acceptable. As shown in Table 1, Cronbach's α for all subscales ranged from 0.71 to 0.91, which indicates good reliability.

**Table 1:** Cronbach's α for the subscales of OLSQ and MSLQ-B.

**Cronbach's** α **for the subscale of OSLQ**		**Cronbach's** α **for the subscale of MSLQ-B**	
**Dependent variable**	**α**	**Dependent variable**	**α**
Goal setting	0.91	Rehearsal	0.82
Environmental structuring	0.86	Elaboration	0.91
Task strategies	0.83	Organization	0.82
Time management	0.88	Critical thinking	0.85
Help-seeking	0.80	Metacognitive self-regulation	0.86
Self-evaluation	0.91	Time and study environment	0.90
		Effort regulation	0.71
		Peer learning	0.77
		Help-seeking	0.73

### 3.2. Descriptive statistics of online learning strategies

The descriptive statistics of OSLQ and MSLQ-B strategies variables for the students that participated in online learning were presented in Table 2. The results showed that different SRL strategies exhibits large differences, but also between students. The highest score of all SRL strategies were consistent with 5, but the lowest score varied greatly from 1 to 2. The mean values ranged from 3.63 to 3.88. The highest average score of learning strategies was environmental structuring, and that of effort regulation was the lowest, which were 4.01 and 3.63, respectively. All SRL strategies also showed large standard deviation (SD) implying a large dispersion of values among individuals. The above analysis showed that there are great differences between different SRL strategies and between different students when accepting the online learning mode during the pandemic.

**Table 2:** Descriptive statistics of online learning strategies.

**Variables**	**Min**	**Max**	**Mean**	**SD**
Goal setting	1.00	5.00	3.86	0.69
Environmental structuring	1.00	5.00	4.01	0.59
Task strategies	1.25	5.00	3.70	0.69
Time management	1.00	5.00	3.81	0.67
Help-seeking	1.00	5.00	3.76	0.64
Self-evaluation	1.00	5.00	3.86	0.74
Rehearsal	2.00	5.00	3.80	0.58
Elaboration	1.17	5.00	3.80	0.60
Organization	1.50	5.00	3.80	0.59
Critical thinking	1.40	5.00	3.74	0.58
Metacognitive self-regulation	2.00	5.00	3.68	0.64
Time and study environment	1.63	5.00	3.88	0.55
Effort regulation	2.00	5.00	3.63	0.63
Peer learning	1.67	5.00	3.71	0.64
Help-seeking	1.50	5.00	3.68	0.55

n = 388.

### 3.3. Differences between GPA-up and GPA-down students in online SRL

**Figure 1:**
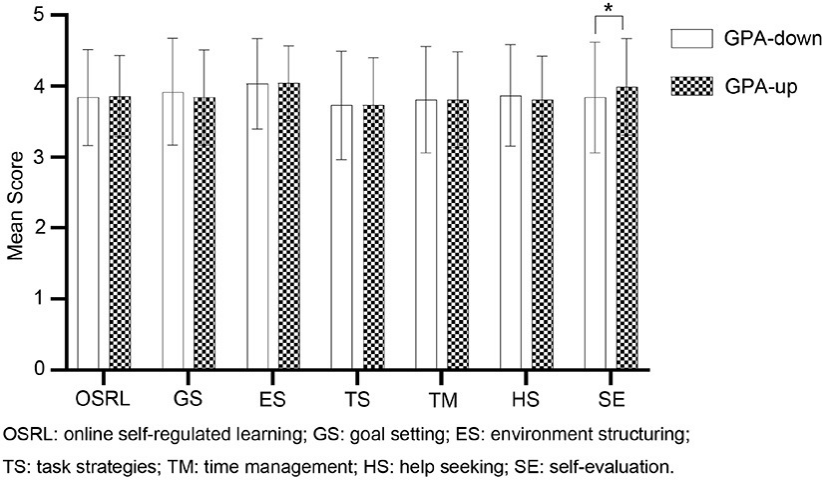
The differential analysis between two sets of samples. White represents GPA-down students based on GPA changes ≤−1.0. The black points represent GPA-up students screened based on GPA changes ≥1.0. Stars represent significant differences between the two groups.

One of the factors that determine the success of online learning is the level of student SRL. Thus, understanding the capabilities of SRL is essential for achieving academic success during this pandemic. Six OSLQ subscales, associated with each of the SRL subprocesses (Barnard et al., 2009), were used (goal setting, environmental structuring, task strategies, time management, help-seeking, and self-evaluation) to analyze the online SRL between GPA-up and GPA-down students. To facilitate the comparison, SRL and all subscale scores were graphed according to different groups (Figure 1). The multi-indicator histogram (independent-sample *t*-test) revealed that all participants reported an average number between 3.7 and 4.1 for both GPA-up and GPA-down students. Environmental structuring got the highest score, while task strategies got the lowest score. In addition, GPA-up students scored significantly greater than GPA-down students on the self-evaluation subscale (*F* = 5.6, *p* < 0.05, with 95% confidence intervals of −0.30 to −0.01). There was no significant difference in other SRL subscales. This finding suggests that self-evaluation relates to students' improved academic performance when studying online.

### 3.4. Differences between GPA-up and GPA-down students in SRL strategies

**Figure 2:**
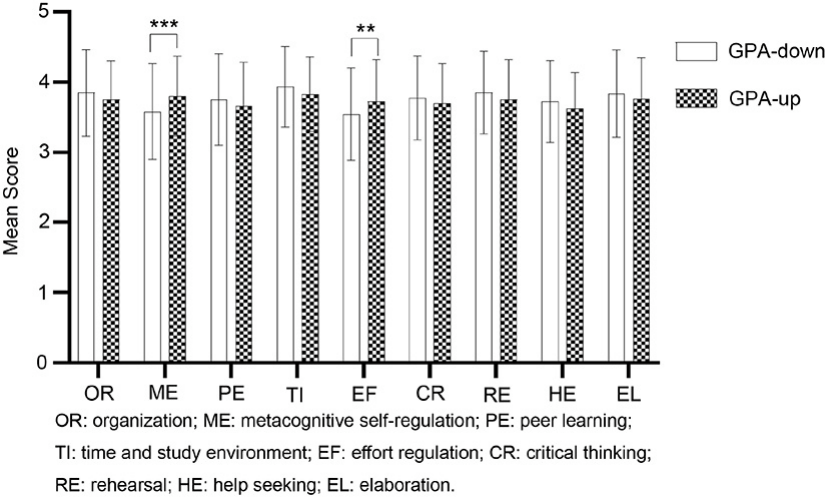
Graphed scores of GPA-up and GPA-down students in the SRL strategies. White indicates GPA-down students, black points indicate GPA-up students, and stars represent significant differences between the two groups. No stars indicate no significant differences in SRL strategies.

In order to further analyze whether there is any difference in self-regulated learning strategies between GPA-up and GPA-down students. The MSLQ-B, including rehearsal, elaboration, organization, critical thinking, metacognitive self-regulation, time and study environment, effort regulation, peer learning, and help-seeking, was used to further analyze the SRL strategies of GPA-up and GPA-down students. Independent-sample t-test was performed using SPSS 22.0. The results revealed that the subscales' scores of the MSLQ-B were between 3.5 and 4.0, which shows a high level of SRL strategies. More importantly, metacognitive self-regulation (*F* = 4.67, *p* < 0.001) and effort regulation (*F* = 0.60, *p* < 0.01) had a significant difference between GPA-up and GPA-down students. As seen in Figure 2, the mean scores of metacognitive self-regulation and effort regulation for GPA-up students were higher than those of GPA-down students significantly. The results also indicated that there are no statistically salient variations among the other SRL strategies. To summarize, metacognitive self-regulation and effort regulation strategies acted as promoters of online academic progress.

### 3.5. The mutual influence effect

**Figure 3:**
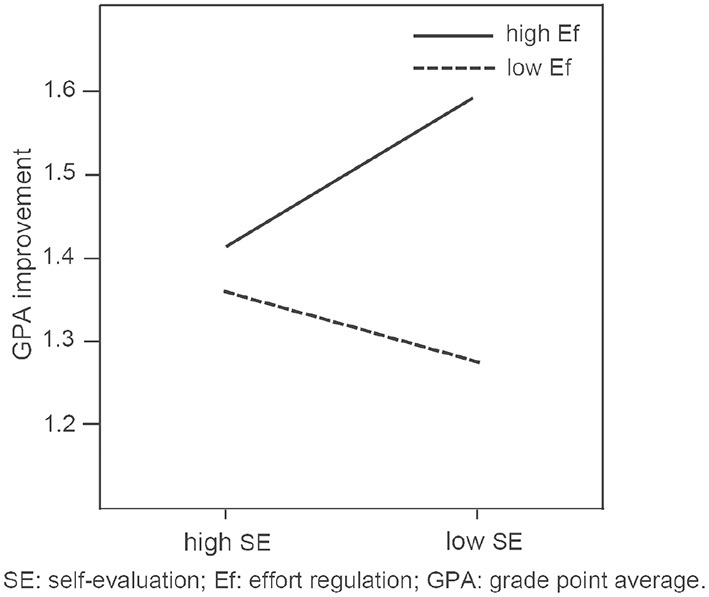
The interaction pattern of SE and Ef on students' GPA improvement.

To examine the mutual influence effect of metacognitive self-regulation, effort regulation, and self-evaluation on the improvement of GPA during online learning, we employed the multivariate analysis of variance. Table 3 and Figure 3 present the mutual influence effect model was significant: *R*^2^ = 0.11, *F* = 3.13, *p* < 0.01. From Table 3, we compared the *F*-value and *p*-value of ME, Ef, SE, ME*Ef, ME*SE, Ef*SE, and ME*Ef*SE interaction, and found that the *F*-value of Ef is the largest (*p* < 0.01). It is concluded that the main effect of effort regulation is significant, while the main effect of metacognitive self-regulation and self-evaluation is not significant. Besides, the interaction effect between effort regulation and self-evaluation is also significant (*F* = 4.59, *p* < 0.05). Figure 3 presents the interaction pattern of SE and Ef on students' GPA improvement during online learning at different SE levels. As the self-evaluation levels increased, the effect of effort regulation on GPA improvement increased for the students with low effort regulation (*F* = 12.51 and *p* < 0.01, with 95% CIs of 0.13–0.46). However, for students with high self-evaluation, there is no significant difference in the improvement of GPA between high effort regulation and low effort regulation.

**Table 3:** The multivariate analysis of ME, Ef, and SE on the improvement of GPA.

**Source**	**Type sun of squares**	**df**	**Mean square**	***F***	***p***
Corrected model	2.114a	7	0.30	3.13	0.004
Intercept	232.06	1	232.06	2406.10	0
ME	0.13	1	0.13	1.32	0.25
Ef	0.90	1	0.90	9.32	0.003
SE	0.03	1	0.03	0.26	0.61
ME*Ef	1.61E-06	1	1.61E-06	0	0.10
ME*SE	0.12	1	0.12	1.19	0.28
Ef*SE	0.44	1	0.44	4.59	0.03
ME*Ef*SE	0	1	0	0.002	0.97
Error	17.17	178	0.10		
Total	370.52	186			
Corrected total	19.28	185			
a.R Squared	0.11				

n = 186; ME, metacognitive self-regulation; Ef, effort regulation; SE, self-evaluation.

## 4. Discussion

In view of the lack of empirical studies in SRL strategies to understand how online learning can be supported, this paper conducted an empirical study on students' learning strategies to further understand the possible ways to promote academic performance in online learning. During the pandemic, universities in China have implemented the online learning mode. By analyzing the SRL strategies of students in online learning, we found that there are great differences between different SRL strategies and between different students when accepting the online learning. Challenges to online learning are generally considered to include accessibility, affordability, flexibility, learning pedagogy, self-regulated learning, and education policy (Murgatroyd, 2021). China has good network infrastructure and digital equipment, and students hardly have equipment and information technology problems. At the same time, the online teaching mode is basically the same as the synchronous or asynchronous live broadcast mode. Therefore, the main influencing factor of academic performance is the difference of students' self-regulated learning ability. The OSLQ and MSLQ-B showed that GPA-up students' self-evaluation, metacognitive self-regulation, and effort regulation were significantly higher than those of GPA-down students. These results suggest that the students who are reflective of their learning behavior and persevere in understanding the learning material despite challenges faced are more likely to achieve academic progress in online learning.

Self-evaluation is a process comprising self-judgments of present performance and self-reactions to these judgments (Brown, 2014). Metacognitive strategies include electing the most effective strategy to approach learning tasks, planning, monitoring one's understanding, and modifying one's learning strategies based on the feedback of learning results (Lai, 2011; Lehmann et al., 2014). Research shows that self-evaluations, reflections, and metacognition are cross-concept relationships, and self-evaluations can help students learn and develop metacognition (Arp, 2016). Thus, the two questionnaires are consistent with each other. The SRL cyclical phases (forethought, performance, and reflection) suggested that every strategies at any phase will have an effect on the subsequent phase (Zimmerman, 1990). Self-evaluation, metacognitive self-regulation, and effort regulation run through the whole cycle of SRL and interact to promote students' academic progress. Some researches focusing on personal characteristics show that self-evaluation is used to explain learners' academic achievements and learning satisfaction. At the same time, self-evaluation is assumed to be a trigger for learners' self-regulation, because people with high self-evaluation are more confident and positive about the results of their efforts, and are more likely to be motivated and participate in learning (Diep et al., 2017). These positive evaluations of individual abilities will stimulate a high degree of motivation to deal with challenges and problems, thus promoting the coping process and improving online learning performance.

Previous research shows that metacognitive strategies contributed the most to academic performance (Goradia and Bugarcic, 2017; Dumford and Miller, 2018). The OSLQ and MSLQ-B results showed that metacognitive strategies also had effect on improving academic performance in the online learning mode during the pandemic, perhaps because learners who engaged in metacognitive strategies were more likely to achieve their goals and engaged more deeply with course assessments. According to Frith, the role of metacognition enables individuals to monitor their current knowledge and skills levels, plan and allocate limited learning resources with optimal efficiency, and evaluate their current learning state (Frith, 2012). However, one study reported that face-to-face teaching practices do not encourage students to use metacognitive strategies (Haidar and Al Naqabi, 2008) because these young adults often still rely on co-regulation from parents or teachers to provide learning orientation (Robson et al., 2020). As learners move to online environments, they may face challenges that they have never encountered before. For example, in an online environment, students' social interaction with teachers is indirect, and there is a lack of effective supervision and evaluation of students' learning (Anthonysamy et al., 2020). Effective metacognitive learners are skilled in self-assessment, recording supervised learning, setting goals, managing time, learning from peers, demonstrating persistence and flexibility, all of which contribute to self-regulated learning (Clark and Dumas, 2016).

Our results also showed that effort regulation plays a significant role in improving academic performance. Effort regulation can be defined as a resource management strategy that refers to the ability to perform even when tasks are viewed as very challenging or uninteresting (Boyraz et al., 2016). Pintrich and Zusho also pointed out that the volitional dimension that determines effort regulation is a key factor in the use of metacognitive strategies (Pintrich and Zusho, 2002). Students with high effort conditioning showed persistence in completing tasks, while students with low effort conditioning were more likely to give up before completing tasks (Richardson et al., 2012). Therefore, effort regulation can be viewed as a necessary precondition for metacognitive self-regulation of learned behavior. In general, online education provides a highly autonomous environment. To be successful, a high degree of self-regulation is essential, while this lack of ability can be compensated for by teacher guidance in a face-to-face classroom (Artino and Stephens, 2009). The online learning environment lacks the effective supervision of teachers, thus, students who can not use this strategy by themselves got poor academic performance.

The mutual influence effect of metacognitive self-regulation, effort regulation, self-evaluation on the improvement of GPA show that effort regulation had the largest main effect. For students with low SE, high Ef can significantly improve their online academic performance. Effort regulation is the embodiment of volition which will enable people to control not only themselves, but also their environment, so as to reduce the obstacles achieving their goals (Kim and Bennekin, 2016). Students who had higher effort regulation have strong volition and motivation, and they are less likely to give up in the face of difficulties.

This study provides evidence that metacognitive self-regulation, effort regulation, and self-evaluation skills in online learning can significantly improve students' academic performance. The results highlights that students should apply these three strategies above in order to increase the likelihood of academic progress in online learning. An important revelation obtained is that educators should consciously exercise students' metacognitive skills, self evaluation, self monitoring in the learning process, which will help students play an important supporting role in future SRL, online learning, and even lifelong learning. Developing students' SRL skills may ultimately be the focus of education in the future.

## 5. Limitations and future research

One shortcoming of this research is that this study was in the middle of a pandemic (fear, uncertainty, etc.) and students' academic performance may be affected by psychological factors. Future research could consider cross-validating our measurements by collecting data from two groups of students at roughly the same time point in a semester. In addition, the learning environment has been noted to influence the way students learn (Severiens et al., 2001). In terms of online contexts, research found that metacognition influences cognitive and emotional engagement, and metacognitive awareness significantly facilitated effective self-regulation (Lehmann et al., 2014; Norman and Furnes, 2016). Future research could focus on comparing whether metacognitive strategies have different effects on academic performance in online and offline learning environments. At the same time, considering that students' SRL ability is a dynamic development process, we can explore how to enhance students' metacognitive skills through online learning, thereby improving students' academic performance. In the later stage, the dynamic change process of students' SRL can be analyzed in depth based on the time axis to find the development mechanism of SRL ability. Finally, although this study demonstrates that some individual SRL strategies are associated with academic performance, autonomous learning strategies are rarely used in isolation. Future research should explore the influence of the combination of learning strategies in a specific environment on SRL.

## 6. Conclusion

In this study, the online SRL questionnaire and learning strategy questionnaire results showed that there are great differences between different SRL strategies and between different students when accepting the online learning during the pandemic. The students whose academic performance is significantly improved in online learning are more capable of using metacognitive strategies, self evaluation, and effort regulation strategies than those whose academic performance is declined. Students with metacognitive skills were indeed able to adjust their learning in the new learning environment and were able to put more effort into regulating the learning process in online learning. This research will help teachers and students establish practices on how to use effort regulation and metacognitive strategies to improve student academic performance, as those students who lack effort regulation and metacognitive strategies may find themselves at a significant disadvantage in online learning (Anthonysamy, 2021).

## Data availability statement

The raw data supporting the conclusions of this article will be made available by the authors, without undue reservation.

## Ethics statement

The studies involving human participants were reviewed and approved by the Ethics Committee of Liaocheng University. The patients/participants provided their written informed consent to participate in this study.

## Author contributions

LX and SP provided the substantial contributions to the research conception and design. LX analyzed and interpreted the data. LX, PD, and CL wrote and revised the paper. All authors contributed to the article and approved the submitted version.

## Funding

This research was partially supported by the Key Teaching Reform Project of Shandong Province (Z2021159), Teaching Reform Project of Liaocheng University (G202119, G202120, G202103, and G201721), Natural Science Foundation of Shandong Province (ZR2016FL13), and Doctoral Research Foundation Project of Liaocheng University (318051532).

## Conflict of interest

The authors declare that the research was conducted in the absence of any commercial or financial relationships that could be construed as a potential conflict of interest.

## Publisher's note

All claims expressed in this article are solely those of the authors and do not necessarily represent those of their affiliated organizations, or those of the publisher, the editors and the reviewers. Any product that may be evaluated in this article, or claim that may be made by its manufacturer, is not guaranteed or endorsed by the publisher.
